# Perinatal diet and offspring anxiety: A scoping review

**DOI:** 10.1515/tnsci-2022-0242

**Published:** 2022-09-06

**Authors:** Sasha Monteiro, Yousef Sadat Nejad, Monique Aucoin

**Affiliations:** Department of Research and Clinical Epidemiology, Canadian College of Naturopathic Medicine, 1255 Sheppard Ave E, Toronto, ON, M2K 1E2, Canada

**Keywords:** nutrition, perinatal, anxiety, mental health, psychiatry

## Abstract

Health behaviors during pregnancy have an impact on the developing offspring. Dietary factors play a role in the development of mental illness: however, less is known about the impact of diet factors during pre-conception, gestation, and lactation on anxiety levels in offspring. This scoping review sought to systematically map the available research involving human and animal subjects to identify nutritional interventions which may have a harmful or protective effect, as well as identify gaps. Studies investigating an association between any perinatal diet pattern or diet constituent and offspring anxiety were included. The number of studies reporting an association with increased or decreased levels of anxiety were counted and presented in figures. A total of 55,914 results were identified as part of a larger scoping review, and 120 articles met the criteria for inclusion. A greater intake of phytochemicals and vitamins were associated with decreased offspring anxiety whereas maternal caloric restriction, protein restriction, reduced omega-3 consumption, and exposure to a high fat diet were associated with higher levels of offspring anxiety. Results were limited by a very large proportion of animal studies. High quality intervention studies involving human subjects are warranted to elucidate the precise dietary factors or constituents that modulate the risk of anxiety in offspring.

## 1 Introduction

A developing offspring is extremely sensitive to their environment which can be impacted by a range of intrauterine exposures and interventions [1]. Health behaviors during pregnancy, such as alcohol consumption and smoking, have long been studied for their adverse effects on offspring development [2]. Emerging evidence shows that many components of maternal diet are critical in shaping aspects of the offspring’s health including the microbiome and neonatal immune system [2]. Although most women are aware that a healthy diet is important during pregnancy, women may lack knowledge, skills, or resources needed to improve their diet [3]. Moreover, healthy eating while pregnant may be a challenge as some women experience food aversions, cravings, nausea, vomiting, or heartburn [3,4]. Evidence from animal studies suggest that maternal diet can change the development of the brain and endocrine system of the developing offspring as well as have a long-term impact on offspring behavior [5]. A balanced, nutritious diet during pregnancy is crucial to maintain the extra demands on the mother’s body [6]. It is established that adequate nutrient intake is needed to support the growth of the fetus and maintain healthy birth weight [6]. There is emerging interest in a possible role of dietary factors on the risk of mental health disorders in offspring as well.

Numerous animal models have been employed to understand the intricate biological mechanisms of developmental programming in maternal exposures and its impact on offspring anxiety [7]. While data from primate studies may be more generalizable to humans, the majority of the research in this area has involved rodent models, in part due to the reduced amount of time for gestation and maturation [7]. Established methods of assessing anxiety behaviors in animal models have been validated. These models have provided insight to the brain and behavioral mechanisms that may be associated with the etiology and physiopathology of anxiety disorders; yet limitations exist with respect to understanding how these findings translate to humans and the emotional experience of anxiety symptoms [8]. Despite limitations, animal models play an important role in understanding the mechanisms contributing to the development of offspring anxiety [9].

Anxiety disorders are common; the lifetime prevalence of any anxiety disorder was reported to be 31.2% in a national comorbidities study [10]. Established risk factors include trauma, stress, genetics, comorbidities, and the use of alcohol and drugs [10]. Anxiety disorders result in a high degree of personal distress, disability, and reduced quality of life among those affected, as well as a societal cost resulting from increased primary care and specialist healthcare service utilization [10,11,12]. The therapeutic approaches commonly provided include psychotherapy and psychopharmacology; [13] however, a substantial number of patients do not find these treatments tolerable, accessible, or effective in relieving anxiety symptoms [14]. To date, strategies for preventing anxiety disorders have consisted primarily of psychosocial interventions directed at children or adolescents of elevated risk of sub-syndromal presentations; however, these require availability of programs and adequate screening to identify individuals at increased risk [15]. Given the limitations in the currently available approaches to treatment and prevention, adjunctive strategies are needed.

The evidence supporting the role of dietary factors in the development and progression of mental illnesses is emerging [16]. Less is known about the impact of perinatal diet factors on anxiety levels in the offspring. A limited amount of synthesis research has identified evidence on particular nutrients or dietary patterns but a systematic effort to capture the range of research that has been undertaken on this topic has not been undertaken [17]. Given the need for additional prevention and treatment strategies, the emerging evidence about nutrition and mental health and the established relationship between perinatal exposures and other offspring health outcomes, research into perinatal diet and offspring anxiety outcomes is warranted. The objective of the present scoping review was to systematically map out the current body of literature on perinatal diet and offspring anxiety to identify nutritional interventions which may be effective for the prevention of offspring anxiety symptoms as well as identify gaps and opportunities for further research.

## 2 Methods

The framework for scoping reviews presented by Arksey and O’Malley provided the methodological approach for this review [18]. A previous, large scoping review was undertaken to assess the relationship between dietary patterns, constituents, and foods in humans and animals, and the development and/or progression of anxiety disorders [19]. This review examined studies involving assessment or administration of a dietary factor and subsequent measurement of anxiety symptoms within subjects [19]. Studies that assessed maternal diet exposure and offspring anxiety levels were excluded from the larger scoping review and have been included in the present review for separate analysis.

The scoping review was completed using an extensive search strategy, and developed *a priori* by an experienced medical librarian (refer Supplemental File 1 in ref. [19] for the complete search strategy). The databases Ovid MEDLINE and Embase Classic + Embase were searched using controlled vocabulary and keywords. The searches were not limited by language or date, but editorials were removed. The searches were performed on March 25th, 2020. The program Abstrackr was used to facilitate title and abstract screening. Screening of abstracts was completed in duplicate, and disagreement was resolved by consensus. Abstracts were screened manually until Abstrackr’s artificial intelligence predicted that all remaining studies were unlikely to be relevant. Testing of this method has found the likelihood of excluding useful studies to be very low [20,21,22].

The present review is an analysis of the human and animal studies related to perinatal diet exposure. Studies were eligible to be included in the present review if they assessed the relationship between perinatal diet and levels of anxiety in the offspring. The perinatal period was defined to include pre-conception, gestation, lactation, and early infancy. Studies were eligible if they assessed anxiety disorders or symptoms using a validated method such as a questionnaire, clinician assessment, or an established method of assessing anxiety severity in animals [23]. Dietary factors of relevance included dietary patterns, macronutrients, micronutrients, individual foods, and natural health products containing nutrients present in the typical North American diet. Studies were ineligible for inclusion if they assessed the use of herbal medicines (apart from those generally used for culinary purposes) or constituents not typically present in the North American diet in appreciable quantities. Review articles, opinion papers, letters, and systematic reviews were all excluded, as well as non-English language papers or inaccessible papers on occasions where the abstract contained insufficient information for data extraction. Eligible study types included observational or clinical studies involving humans or animals as well as meta-analyses. Review articles, opinions, and systematic reviews were excluded.

In addition to reviewing the results identified by the previous scoping review, a grey literature search was also undertaken. This involved the search of two databases for systematic reviews on related topics and review of the included studies within each review. Any studies that met the criteria for the present review but had not been previously identified were included. A piloted extraction template was used for data extraction, in duplicate. Studies were sorted based on similar diet interventions and whether the study increased or decreased the dietary factor of interest. Studies reporting an increase or decrease in offspring anxiety were counted and presented in figures.

## 3 Results

**Figure 1:**
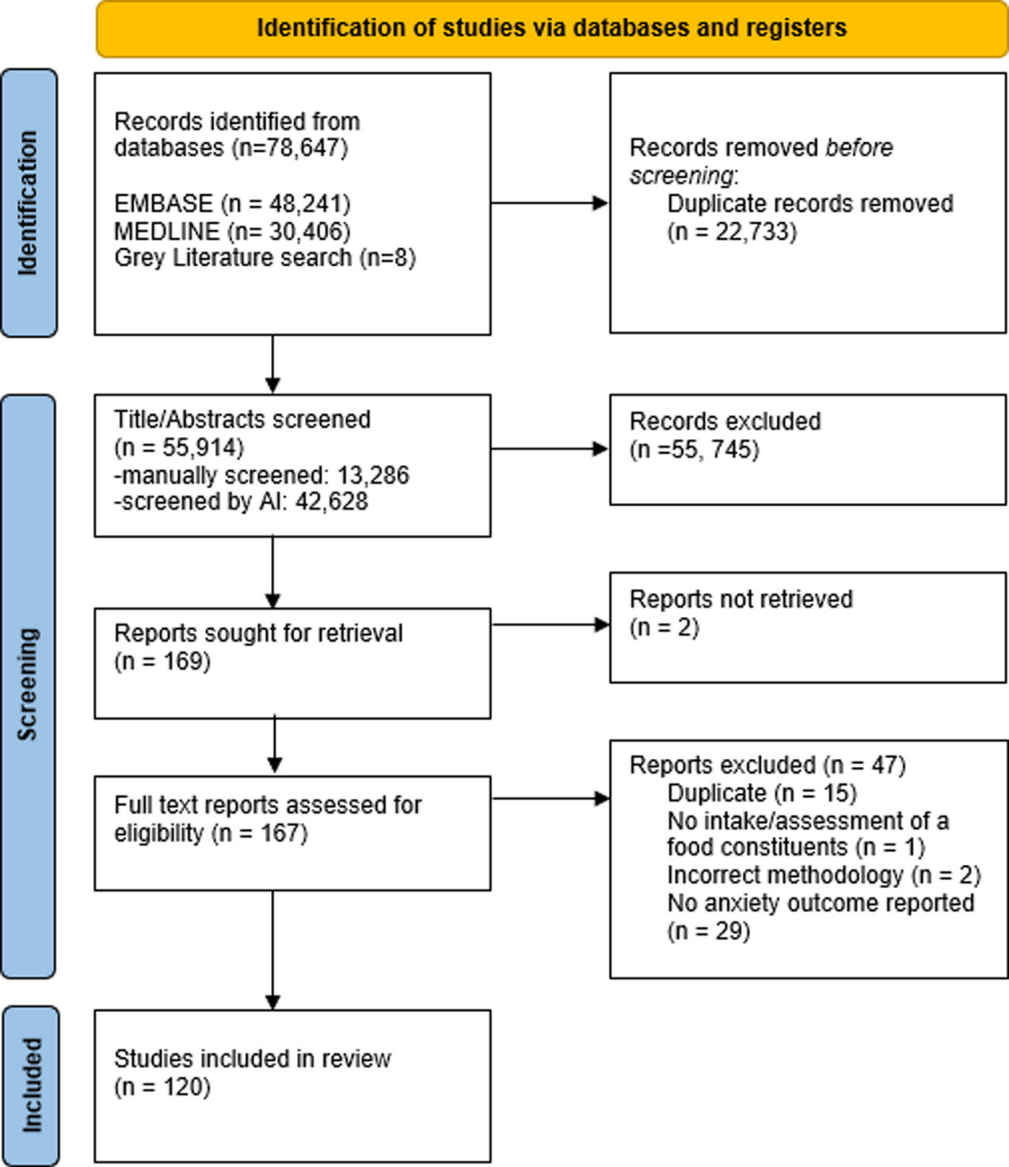
PRISMA flow diagram.

The larger scoping review search identified 55,914 unique results (Figure 1). Eight additional articles were identified through the grey literature search. Manual screening of the titles and abstracts was completed for 13,286 results. The remaining results (*n* = 42,628) were screened by Abstrackr’s artificial intelligence feature.

After title and abstract screening for the present sub-analysis on perinatal studies, six human studies were included. Following full text screening, three met criteria for inclusion, two observational studies and one experimental study. After title and abstract screening, 169 animal studies met criteria. Following full text screening, 120 met criteria for inclusion in the analysis. Of these, one was a meta-analysis. The vast majority of the studies used rodent models (*n* = 117, 98%) with the remaining 3 studies involving human participants. A list of all studies included is available in Supplemental File 1.

### 3.1 Human studies

There is a very small body of literature that exists in the form of human experimental (*n* = 1) and observational (*n* = 2) studies that sought to modify or observe perinatal intake of dietary constituents and study anxiety outcomes in offspring. One experimental study aimed to assess the effects of perinatal food supplementation on school-aged children’s behavior [24]. Mothers from a malnourished population were recruited and given varying amounts of food supplementation (low or high) pre- and postnatally and their offspring (78 boys and 60 girls) were observed [24]. The high supplementation participants were given a mixture of vegetable protein, dry skim milk, and sugar, whereas the low supplementation participants were given a calorie-only supplement of only sugar and flavoring. One cup (180 mL) of the high supplement contained 11.5 g of protein and 163 calories. One cup of the low calorie-only supplement contained 59 calories. Both groups received supplements which contained essential vitamins and minerals [25]. Children were then put into same-sex, six-person groups and observed over a 2 day period [25]. Children took part in approximately nine different situations including both free play activities (e.g., clay construction and painting a mural) and structured situations that would mimic the type of challenges or stresses encountered routinely in everyday life [25]. Children born to highly supplemented mothers demonstrated superiority on responses to moderately stressful situations when compared to children born to mothers in the low supplementation group [24]. Moreover, when engaging with peers, children of highly supplemented mothers showed more positive affect and were less anxious [24].

Of the observational studies reporting on the relationship between dietary exposure and anxiety outcomes, one retrospective study enrolled 77 adults who experienced protein malnutrition within their first year of life followed by good health and nutrition for up to 12 years [26]. When compared to healthy controls, early-life malnourished participants experienced more anxiety and a lower sense of efficacy and competence as assessed by the NEO-PI-R, a 240 item self-report questionnaire [26]. Another retrospective observational study assessed the effects of maternal caffeine consumption, in the form of coffee and tea, between 15 and 30 weeks of gestation on offspring anxiety [27]. Women were interviewed twice during pregnancy (15–30 weeks of gestation) and twice following delivery (when the child was 6–18 months of age). Measured covariates included maternal age at birth, maternal pre-pregnancy body mass index, maternal smoking during pregnancy, maternal socioeconomic status, marital status, birth year of offspring, and sex of offspring [27]. 11 years later, data were collected in the form of the Strengths and Difficulties questionnaire by the children, parents, and their teachers. [27] High maternal caffeine consumption (more than 8 cups/day) at 15 weeks of gestations resulted in increased severity of anxiety disorders in 11-year-old children [27]. These findings support the hypothesis that high caffeine consumption during pregnancy may affect the fetal brain and program for behavioral disorders later in life; however, confounding factors such as genetics, dietary intake and/or socioeconomic status may be accountable for the observed associations [27]. While these human studies suggest that there may be an association between perinatal diet exposure and offspring anxiety levels, the quantity of data is very limited; as such an exploration of other forms of evidence on this topic is warranted.

### 3.2 Animal studies

**Figure 2:**
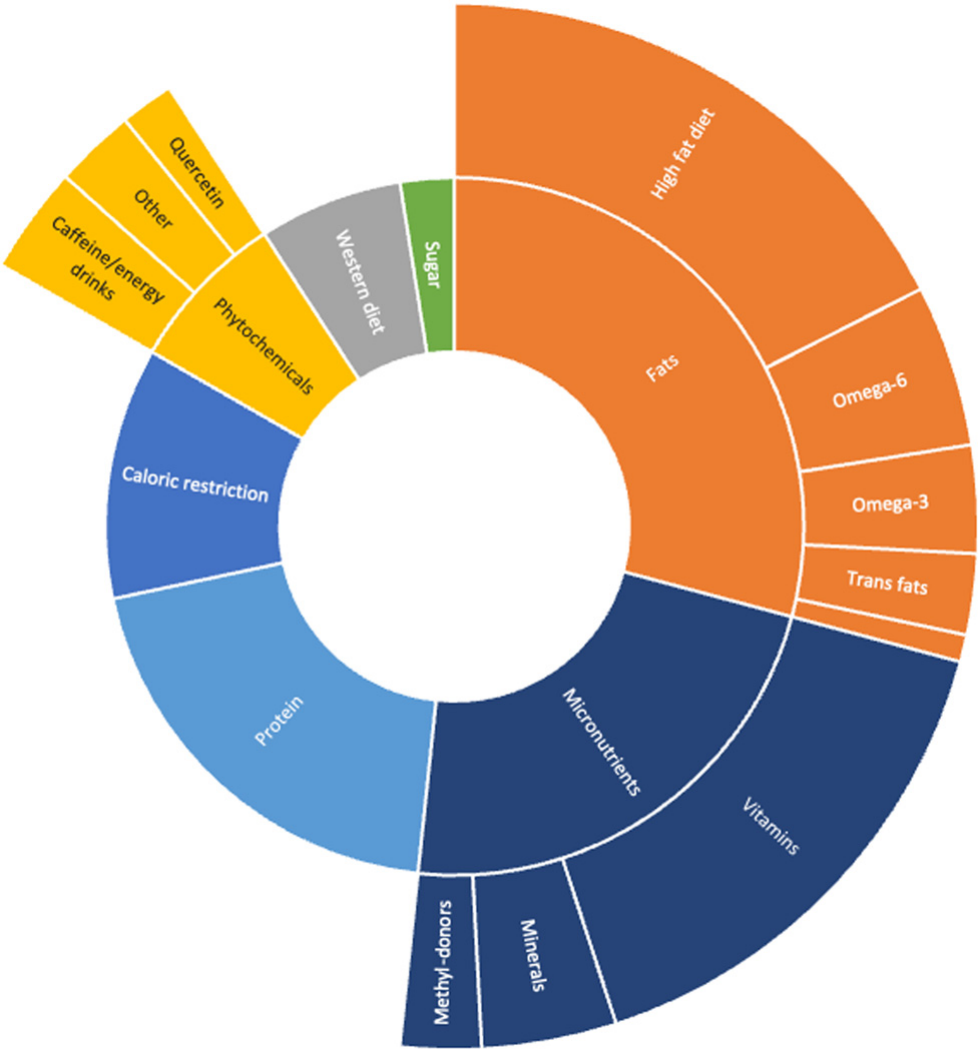
Distribution of included animal studies by intervention.

**Figure 3:**
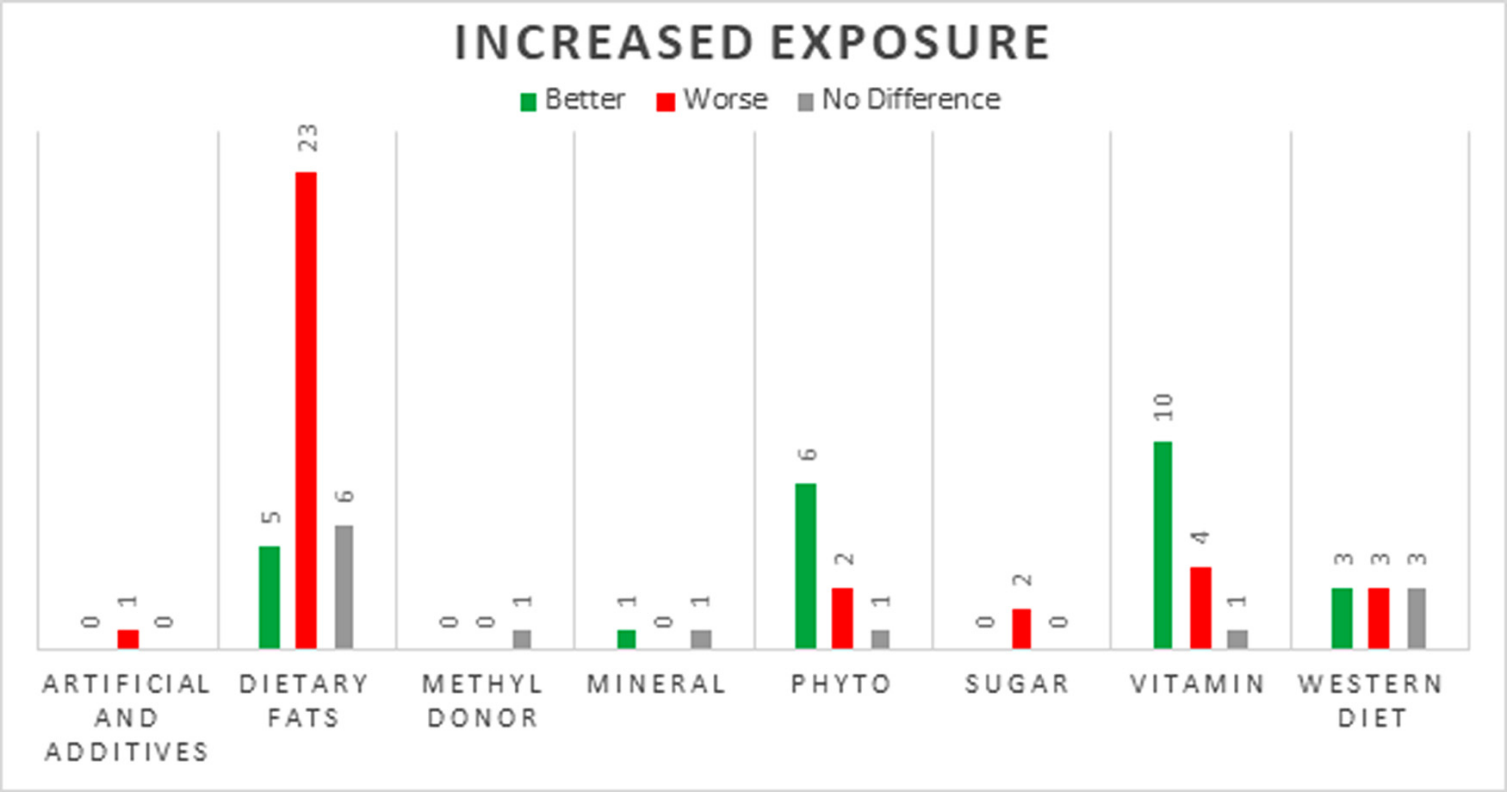
Change in anxiety levels in animals in response to an increase in exposure to dietary factor.

**Figure 4:**
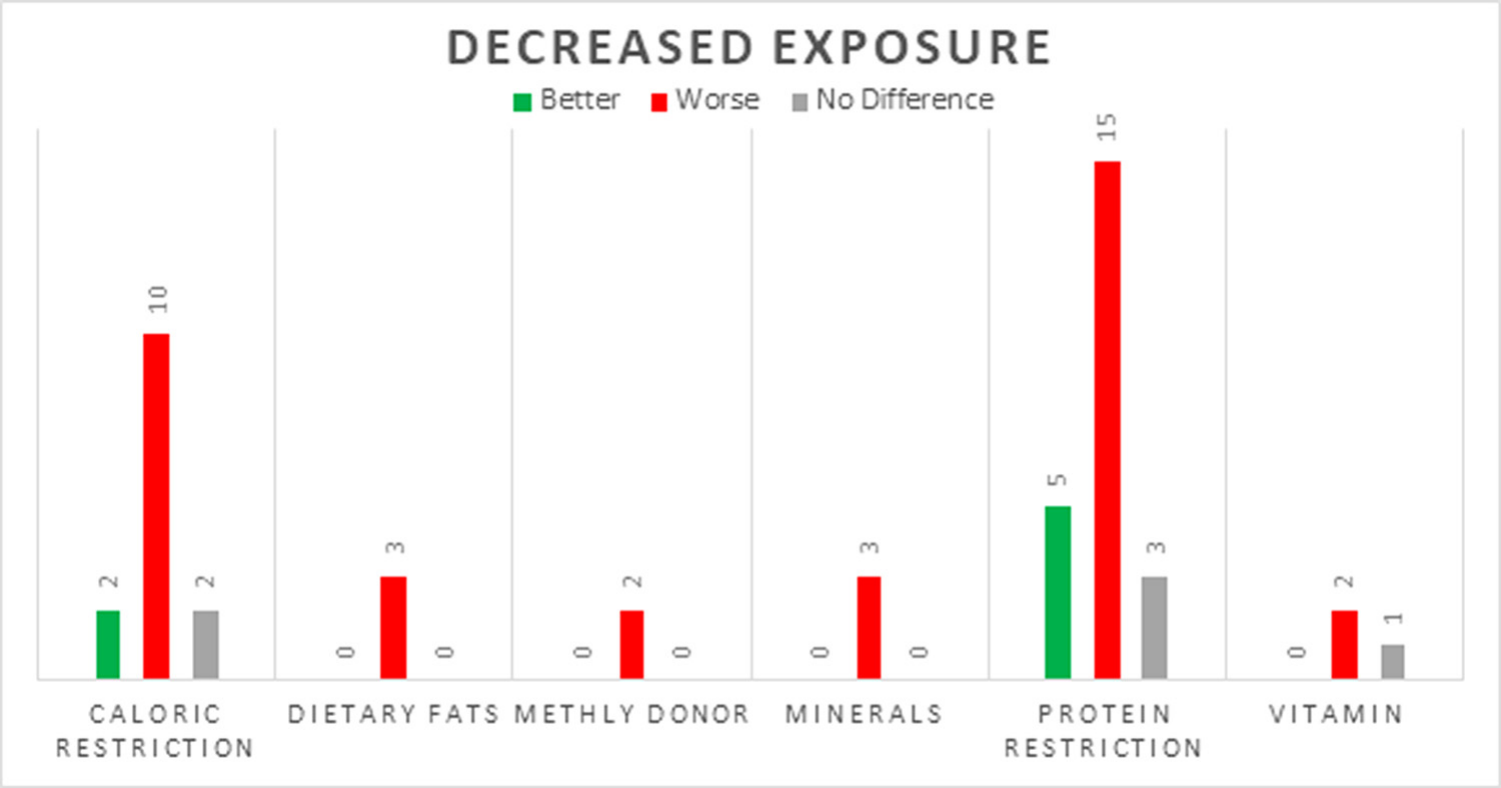
Change in anxiety levels in animals in response to a decrease in exposure to dietary factor.

Numerous studies included in this review assessed the impact of exposure to different dietary patterns and constituents on offspring anxiety levels in animal models (Figure 2). In animal studies, anxiety-like behavior is assessed by measuring the subject’s response to a novel and potentially threatening environment using batteries of tests. Among them, the elevated plus maze test is the most common and simple method to assess anxiety in rodents [23]. Other tests included the light–dark box, social interaction test, the open field test, and the novelty suppressed feeding test [23,28]. Figures 3 and 4 present the number of animal studies showing an association between changes in a dietary variable and anxiety outcomes.

A 2016 meta-analysis included 46 animal studies that measured the effects of caloric restriction, protein restriction, and overfeeding around the period of gestation [29]. Despite the high heterogeneity among studies, the results did not indicate a difference in anxiety symptoms within offspring [29].

#### 3.2.1 Dietary fats

A typical rodent diet contains about 10% fat, while diets containing 45–60% fat are considered “high-fat” [30]. Numerous animal studies have investigated the effects of increased intake of fat on rodent models of anxiety [31,32,33,34,35,36,37,38,39,40,41,42,43,44,45,46,47]. Many animal studies (*n* = 15, 68%) have reported an increase in anxiety behaviors in response to consumption of a high fat diet [37,38,39,40,41,42,43,44,45,46,47,48,49,50,51]. Opposite effects were seen when levels of omega-3 and omega-6 fatty acids were manipulated. An increase in omega-6 primarily worsened anxiety (*n* = 5, 71%) [52,53,54,55,56], whereas a decreased exposure of omega-3, worsened anxiety (*n* = 3, 100%) [57,58,59]. A smaller number have reported a negative effect from diets high in trans-fats (*n* = 2, 67%) [47,60].

#### 3.2.2 Methyl-donors, minerals, and vitamins

A very small body of evidence (*n* = 3) assessed the impact of dietary factors involved in methylation on anxiety with 67% showing an increase in anxiety when rodents were fed a diet depleted in methyl donors including choline, folate, and methionine [61,62,63]. One study that increased daily choline intake to 1 g found no difference in the anxiety symptoms [61].

The minerals that have been assessed for an impact on offspring anxiety symptoms most frequently are zinc, magnesium, iron, and selenium. Three studies (60%) suggest that when these minerals are decreased or eliminated from the maternal diet, there is an increase in offspring anxiety behavior [64,65,66]. A single study examining the impact of increased zinc reported no difference in the anxiety symptoms [67]. Only one study looked at the impact of selenium and suggested an anti-anxiety effect from supplementing with this nutrient [68].

A large number of animal studies have investigated the effects of vitamins B, C, D, E, choline, and folic acid with primarily beneficial effect on offspring anxiety symptoms [61,69,70,71,72,73,74,75,76,77,78,79,80,81,82,83,84]. Among the studies that increased vitamin exposure, two thirds (*n* = 10, 67%) of the studies were associated with less anxiety symptoms [69,70,71,72,73,74,75,76,84], while one quarter showed an increased anxiety (*n* = 4, 27%) [61,78,79,80], and only one study reported no difference [77]. Of these studies, a diverse range of animal models were used. One involved the administration of choline to a mouse model of autism and found that high choline intake (36 mmol kg^−1^) notably reduced anxiety behavior [71]. Another study evaluated a wide range of perinatal vitamin D3 exposure; both deficiency and excess supplementation resulted in higher levels of anxiety during the juvenile period, when compared to adequate supplementation [75]. The remaining four studies decreased vitamin exposure and reported increased levels of anxiety [75,81,82,83].

### 3.3 Protein restriction

A large number of studies (*n* = 20) investigated the effects of different levels of dietary protein on anxiety symptoms [79,85,86,87,88,89,90,91,92,93,94,95,96,97,98,99,100,101,102,103]. More than two thirds (*n* = 14, 70%) of animal studies assessing the impact of protein malnutrition reported a worsening of anxiety symptoms [79,85,90,91,92,93,94,95,96,97,98,99,100,101]. Five studies (23%) found that reducing protein decreased anxiety symptoms [86,87,104,105,106], and 2 (9%) found no difference [88,89]. One study reported mixed findings when mice were fed a low protein diet prenatally and a high fat diet postnatally; naturally conceived mice showed a modest increase in anxiety, whereas *in vitro* fertilized C57B1/6J mice showed a decrease in anxiety behavior [103].

### 3.4 Caloric restriction

Many animal studies (*n* = 14) have investigated the effects of decreased intake of calories on rodent models of anxiety [23,29,88,107–117]. Ten studies (71%) reported an increase in anxiety behaviors in response to consumption of less calories, [23,109–117] 2 (14%) showed a decrease in anxiety [107,108] and 2 (14%) showed no difference [29,88]. One meta-analysis, using different inclusion criteria compared with the present review, combined the results of 55 studies in which researchers restricted both calories and proteins and found that there was no difference in the anxiety symptoms [29]. The degree of caloric restriction varied widely with most studies restricting calories by 25–80%. Two studies that found an improvement in anxiety had a caloric reduction of 25–30%, [107,108] while all of the studies that restricted calories by more than 50% reported increased anxiety symptoms [23,109–115].

#### 3.4.1 Phytochemicals

The results of the studies related to phytochemicals were largely positive. One study looked at the effect of green tea and found that administration of higher doses (20–50 g L^−1^) resulted in an anxiolytic effect [118]. Anti-anxiety effects have also been reported following administration of fruit juice (*n* = 1) and quercetin (*n* = 2) [119–121]. A small number of studies (*n* = 3) have explored the effects of caffeine on anxiety symptoms in rodents; one reported an increase in anxiety with a higher intake of maternal caffeine (*n* = 1, 33%) [122].

#### 3.4.2 Western diet

The “Western” diet typically includes higher intake of sugar, protein, and fat, and low intake of fruit, grains, and vegetables. In many studies, the diet was compared to a chow diet, that was composed of agricultural by-products, which included ground wheat, oats, soybean, a protein source, and a vegetable oil. The results of the studies assessing the impact of a Western diet on offspring anxiety symptoms are not consistent in their findings. Out of the eight studies that studied the effects of perinatal exposure to the Western diet, three reported benefit, [123–125] three reported a worsening in anxiety, [126–128] and two showed no difference [127,129].

#### 3.4.3 Sugar

A small body of literature (*n* = 3, 67%) suggests a possible connection between increased sugar intake and worsened offspring anxiety symptoms [130–132]. The sugars and sweeteners that were assessed for an impact on anxiety symptoms included sucrose, fructose, and aspartame. Of the three studies, two found worsened anxiety and the remaining one reported mixed results; consumption of a high fructose diet showed an overall increase in anxiety in juvenile rats whereas it showed an overall decrease in adults [130].

#### 3.4.4 Microbiome

A very small number of studies (*n* = 2) have explored the impact of prebiotic and probiotic supplementation on animal models [133,134]. Both animal trials reported an improvement in anxiety symptoms when mice were supplemented with lactobacillus or a combination of short- chain galacto-oligosaccharides and long-chain fructo-oligosaccharides [133,134].

#### 3.4.5 Other

There was only one study that looked at the effects of an artificial food colorings and additives (AFCAs) on anxiety outcomes [135]. Prenatal exposure to commonly used AFCA, specifically consisting of erythrosine, was associated with increased levels of offspring anxiety [135].

## 4 Discussion

The findings of the present scoping review suggest that exposure to certain dietary patterns and constituents during the perinatal period may be associated with an increased or decreased risk of the development of anxiety disorders in offspring. Predominantly, an increase in anxiety were seen following caloric restriction, protein restriction, reduced omega-3 consumption, and exposure to a high fat diet. Conversely, increased consumption of phytochemicals and vitamins were predominantly associated with a decrease in anxiety symptoms. Human studies looked at the impact of protein restriction, maternal caffeine consumption, and undernutrition. In a population experiencing high rates of malnutrition, nutritional supplementation intervention was associated with a decreased risk of anxiety in the offspring [26]. Similarly, Barrett et al. found positive effects from nutritional supplementation on reducing offspring anxiety within an endemically malnourished population [24]. Given the limited scope of research, further human studies are needed.

### 4.1 Anxiety pathophysiology

While the exact pathophysiology of anxiety disorders is not fully understood, many mechanisms have been identified. Dysregulation of several regions of the brain have been implicated in the development of anxiety disorders, particularly structures in the amygdala, which are more active in anxious individuals compared with healthy controls [136]. The prefrontal cortex regulates anxiety through an impact on amygdala activity and has been found to be hypoactive in anxious individuals [137]. With respect to neurochemical balance, several neurotransmitters have been implicated in the pathophysiology of anxiety disorders. Dysregulation of gamma-aminobutyric acid, serotonin, endocannabinoids, and opioid peptides have been identified among anxious individuals [138]. High levels of anxiety symptoms have been associated with altered functioning of the autonomic nervous system and the hypothalamic-pituitary axis [139].

In addition to these established mechanisms, there is building evidence that inflammation may play a role in mental illness [140]. Cytokines are communication molecules released by the immune system which modulate a wide range of body systems, including the nervous system, through pro- or anti-inflammatory effects [141]. In response to chronic inflammatory challenge, this process may contribute to the development of neuropsychiatric symptoms [140]. Animal models that are designed to overexpress pro-inflammatory cytokines develop anxious behavior [142]. Higher levels of these molecules have been reported in humans with higher anxiety and experimental studies involving administration of pro-inflammatory cytokines to healthy individuals have reported an increase in anxiety [143,144]. Another recent area of study in psychiatry is the impact of the microbiome on mental wellbeing. There is evidence that individuals with anxiety disorders have alterations to their microbiome composition and that negative changes in the microbiome alter the function of the hypothalamus [145]. These contributing pathways may help to understand the processes by which dietary exposures impact the development of psychiatric symptoms in the present data.

### 4.2 Dietary fats

Dietary fats play a role in important physiologic processes; however, consumption of excess amounts and certain types have been associated with harm [146]. Given the high level of dietary fat consumption and maternal obesity in developed nations, these findings have crucial implications for the mental health of future generations [2]. Overall, animal evidence shows that supplemental omega-6 fatty acids worsen anxiety symptoms. Whereas a diet containing adequate omega-3 fatty acids may have anti-anxiety effects [147]. A possible mechanism to explain this response is the role of inflammation in the development of psychiatric disorders, including anxiety [147]. Intake of omega-3 fatty acids has been shown to increase the production of anti- inflammatory cytokines thereby reducing levels of inflammation, whereas omega-6 fatty acids increase levels of inflammatory through cytokine production [140]. There is noteworthy evidence also to suggest that a diet high in total fat and trans-fat may worsen anxiety symptoms in offspring. In contrast, one study assessing the ketogenic diet, a diet that is extremely low in carbohydrates and generally high in fat content, suggested a possible protective effect with respect to offspring anxiety [31]. One possible explanation for these seemingly contradictory results may be the type of fat that was used in the interventions. Many of the studies delivering a high fat diet primarily delivered sources of saturated fat or omega-6 fatty acids with the intention of inducing obesity or metabolic dysregulation [37,42]. While the ketogenic diet study included in this review did not report the type of fat used in the diet, it is hypothesized that the type of fat or the inclusion of other constituents may differ. Similarly, variety in the type of fatty acids used to supplement the high fat diets may be responsible for the variation in the outcomes reported. Many studies failed to report the type of fat administered; more transparent reporting of the type of fat used in future animal studies is needed. Overall, the type of dietary fat may be a significant factor in addition to the quantity of fat consumed to determine impact on offspring anxiety.

### 4.3 Protein restriction

This review identified some evidence of an association between protein restriction and worse offspring anxiety symptoms. This macronutrient, made up of amino acid subunits, is crucial for the growth and production of cellular receptors, transporters, and signaling molecules like neurotransmitters [9]. Nine essential amino acids cannot be produced endogenously and are obtained from the diet. Research in animal models has shown that placental amino acid transport is decreased when protein restriction is limited due to maternal amino acid availability [148]. Given the increased attention to plant-based diets, which may be low in protein, [149] further research on the effects of protein deficiency is warranted. It is noted that a smaller number of animal studies reported an improvement in anxiety symptoms among animals exposed to perinatal protein restriction. Authors of one such study hypothesize that the decreased behavioral inhibition seen in these animal subjects may be due to higher impulsiveness; this trait is a characteristic of attention deficit disorder which is known to be more common among malnourished children [85,106]. As such, hypotheses about the benefits of protein restriction warrant caution.

### 4.4 Caloric restriction

The findings of studies modulating the energy intake of the subject suggest that the relationship between caloric restriction and anxiety may be complex. In some, but not all of the included studies, a reduction of 20–40% of calories along with all necessary nutrients was associated with decreased anxiety. Overall, caloric restriction has the potential to prevent several diseases by utilizing protective functions that increase longevity in animal models [150]. Although the exact mechanism is still unclear, it is thought that mice exposed to moderate caloric restriction have attenuated inflammation, improvement in glucose metabolism, activation of anti-inflammatory pathways, and improvement in hippocampal plasticity [151]. On the other hand, severe maternal restriction of 40% or more can cause metabolic dysfunction, resulting in an increased adiposity and altered HPA axis [43]. It is important to note that there were differences in timing of caloric restriction between the included studies which could have impacted the study outcomes. Preliminary evidence suggests that restriction at different periods of gestation can have a unique effect on offspring anxiety; however, more research is warranted to confirm this hypothesis [93].

The mechanism by which severe caloric restriction may be associated with harm may be related to the established connection between maternal diet and neurological, immunological, and central nervous system development of offspring; disruption of any of these systems due to malnutrition can play a role in the development of mental disorders and fetal development [152]. During pregnancy, maternal malnutrition hinders placentation, impacting placental size, morphology, and blood flow all of which decrease the availability of nutrients to the fetus; this vulnerable fetal nutrition status has been linked to impacts on organogenesis, physical growth, emotional development, and morbidity [153]. The findings of undernutrition are consistent with previously published literature which report early undernutrition can have negative long-lasting impact on children’s development and anxiety levels [154].

### 4.5 Methyl-donors, minerals, and vitamins

Micronutrients like vitamins and minerals play a role in a large number of physiologic processes which may impact mental health. In this review, animal data showed anxiolytic benefits of greater vitamin and mineral intake. Specifically, an increase in choline, vitamin B3, D, and E showed a reduction in anxiety whereas an increase in folic acid worsened anxiety in several studies [78,80,155]. Choline, especially important during pregnancy, has a variety of benefits and has been shown to play a role in the development and treatment of mental health disorders [156]. B vitamins and folic acid contribute to the methylation balance which is hypothesized to be relevant to the pathophysiology of psychiatric illnesses [25]. However, very high doses of folic acid were shown to be harmful to the brain development of the fetus hypothesized by the metabolism of the body. There has been a rise in research focusing on the potential role of vitamin D3 deficiency in mental health [157]. Research to date shows that maternal vitamin D3 deficiency especially in the later stages of gestation can produce behavioral changes in offspring [158]. Due to the increased demands of physiological changes in pregnancy, micronutrient deficiency can be exaggerated and as such can result in offspring complication [159].

### 4.6 Sugar

A small but relatively consistent body of research suggests an association between higher sugar intake and higher offspring anxiety. High sugar consumption in adults results in an increase in blood glucose levels; however, a significant compensatory insulin spike may result in reactive hypoglycemia [160]. The hypoglycemic response has been correlated with an acute rise in epinephrine which contributes to neuropsychiatric symptoms including anxiety [160]. Dysregulation of blood sugar levels may also impact offspring mental health. It has been hypothesized that high sugar intake during the perinatal period could predispose offspring to substance use disorders [161]. Research on the effects of sugar intake in the perinatal period is a significant gap and warrants further study.

### 4.7 Microbiome

Although only a small number of studies looked at the impact of prebiotic and probiotic supplementation on animal models, preliminary evidence suggests an improvement in anxiety symptoms with supplementation of lactobacillus, short-chain Galacto-oligosaccharides, and long- chain Fructo-oligosaccharides [133,134]. Dysbiosis and inflammation of the gut have been associated with several psychiatric illnesses, whereas a modulation of the production of gut peptide involved in the gut-brain axis and neurotransmitter synthesis has been linked to a reduction in the anxiety symptoms [162].

### 4.8 Strengths and limitations

The present review had a number of strengths. An extensive search strategy was employed. Inclusion criteria were determined *a priori* and screening occurred in duplicate. There were also limitations. The extensive search strategy resulted in a very large number of results which necessitated the use of the software program Abstrackr. While previous investigations suggest a low likelihood of relevant studies being missed, it is possible that this may have occurred, resulting in the omission of relevant studies [20,21,22]. The broad scope of the research question meant that studies related to a wide range of diet exposures were reviewed. Given the large volume, in depth analysis, including assessment of the quality of individual studies was not undertaken. Consequently, the results of the review may include over-simplifications. Another limitation was the heterogeneity among individual studies assessing the same dietary patterns. Studies termed “high fat” supplemented a variety of dietary fats, including omega-3, omega-6, lard, and olive oil; however, in many studies a description of the specific fatty acids used was absent. The effect of different dietary fats on health outcomes can vary widely and thus simply describing diets as low or high in fat could result in inconsistent findings.

The most significant limitation of this review, which precludes the application of results to clinical care, is that the vast majority of the studies involved animal subjects rather than humans. Animal models have several advantages for the study of perinatal nutrition such as a short gestation period and lifespan. These trial designs allow for highly controlled manipulation of the diet which is rarely possible in human trials. Further, ethical concerns about exposing pregnant humans to potentially harmful substances or withholding essential nutrients prevent these trials from taking place. However, the applicability of these animal study findings to humans are unclear. Assessment of anxiety symptoms is challenging as animals can not directly communicate with researchers about their emotional state. Instead, several behavioral tests have been designed and validated, allowing researchers to quantify and compare the effects of different environmental exposures on anxiety symptoms in animals [163]. Many of the models measure coping mechanisms, an animal’s attempt to avoid harm or distress. Many rely on assessment of exploratory behavior, social behavior, conflict tests, and avoidance tests. These tests have been validated and are considered acceptable ways to measure anxiety in animal research [163]. However, given the differences in physiology and psychology between animals and humans, the findings of animal studies may not be reproducible in humans.

### 4.9 Implications of findings and conclusion

Overall, there is preliminary evidence that dietary exposures during the perinatal period may impact offspring anxiety levels. At this time, the research on this topic is limited to primarily preclinical research and is highly heterogenous with respect to the interventions provided to study subjects; as such a systematic review on this topic is not currently warranted. Given the preliminary evidence of a trend toward worse offspring anxiety, there is a need for further research on the effects of material dietary fat intake with increased attention to and transparent reporting of the type of fat consumed by study subjects. Further study on the effects of protein and caloric restriction are also warranted with particular attention to the degree of restriction and the timing with the gestational period. Further research is warranted on the effects of vitamin and mineral intake. Perinatal sugar intake is an understudied area that warrants further investigation.

Overall, future research should involve human subjects in order to understand how the associations observed in animal studies might translate to human health. This could include prospective observational studies of potentially harmful diet patterns or clinical trials assessing diet patterns that are hypothesized to provide benefit. The findings of these future studies could inform public health initiatives in order to play a role in the prevention of anxiety symptoms and disorders and decrease the population burden of these common conditions. While overall the evidence is preliminary and limited, the associations identified in this review are consistent with generally accepted diet recommendations. Despite the need for more research in this area, this review highlights the need to adequate nutrition during the perinatal period.

## Supplementary Material

Supplementary Material
