# Supplementary material for: Perinatal diet and offspring anxiety: A scoping review
Source: Transl Neurosci. 2022 Sep 6;13(1):275–90. doi: 10.1515/tnsci-2022-0242 (PMC9449687; doi:10.1515/tnsci-2022-0242)
Supplement: Supplementary Material [file tnsci-2022-0242-sm.pdf]

Supplementary material

Study citation	Intervention category	Increased or decreased exposure	Change in anxiety
Doguc DK, Aylak F, Ilhan I, Kulac E, Gultekin F. Are there any remarkable effects of prenatal exposure to food colourings on neurobehaviour and learning process in rat offspring? Nutr Neurosci. 2015;18(1):12-21.	Artificial Substances and Food Additives	Increased	Worse
Akitake Y, Katsuragi S, Hosokawa M, Mishima K, Ikeda T, Miyazato M, et al. Moderate maternal food restriction in mice impairs physical growth, behavior, and neurodevelopment of offspring. Nutr Res. 2015;35(1):76-87.	Caloric Restriction	Decreased	Worse
Barrett DE, Radke-Yarrow M. Effects of nutritional supplementation on children's responses to novel, frustrating, and competitive situations. The American Journal of Clinical Nutrition. 1985;42(1):102-20.	Caloric Restriction	Increased<br>Decrease	Better<br>Worse
Besson AA, Lagisz M, Senior AM, Hector KL, Nakagawa S. Effect of maternal diet on offspring coping styles in rodents: a systematic review and meta-analysis. Biol Rev Camb Philos Soc. 2016;91(4):1065-80.	Caloric Restriction<br>Protein Restriction	Decreased	No difference
Fernandez S, Gonzalez C, Patterson AM. Oil enriched diets and behavioral parameters in rats' recovery from early undernutrition. Physiol Behav. 1997;62(1):113-9.	Caloric Restriction	Decreased	Worse
Fisch J, Feistauer V, de Moura AC, Silva AO, Bollis V, Porawski M, et al. Maternal feeding associated to post-weaning diet affects metabolic and behavioral parameters in female offspring. Physiol Behav. 2019;204:162-7.	Caloric Restriction	Decreased	Better
Govic A, Bell V, Samuel A, Penman J, Paolini AG. Calorie restriction and corticosterone elevation during lactation can each modulate adult male fear and anxiety-like behaviour. Horm Behav. 2014;66(4):591-601.	Caloric Restriction	Decreased	Worse
Govic A, Penman J, Tammer AH, Paolini AG. Paternal calorie restriction prior to conception alters anxiety-like behavior of the adult rat progeny. Psychoneuroendocrinology. 2016;64:1-11.	Caloric Restriction	Decreased	Better
He B, Xu D, Zhang C, Zhang L, Wang H. Prenatal food restriction induces neurobehavioral abnormalities in adult female offspring rats and alters intrauterine programming. Toxicol Res (Camb). 2018;7(2):293-306.	Caloric Restriction	Decreased	Worse
Jaiswal AK, Upadhyay SN, Satyan KS, Bhattacharya SK. Behavioural effects of prenatal and postnatal undernutrition in rats. Indian J Exp Biol. 1996;34(12):1216-9.	Caloric Restriction	Decreased	Worse
Jaiswal AK, Upadhyay SN, Satyan KS, Bhattacharya SK. Comparative effects of prenatal and postnatal undernutrition	Caloric Restriction	Decreased	Worse

(*Continued*)

(*Continued*)

on learning and memory in rats. Indian J Exp Biol. 1999;37(1):17-22.			
Kumon M, Yamamoto K, Takahashi A, Wada K, Wada E. Maternal dietary restriction during lactation influences postnatal growth and behavior in the offspring of mice. Neurochemistry International. 2010;57(1):43-50.	Caloric Restriction	Decreased	Worse
Levay EA, Paolini AG, Govic A, Hazi A, Penman J, Kent S. Anxiety-like behaviour in adult rats perinatally exposed to maternal calorie restriction. Behavioural Brain Research. 2008;191(2):164-72.	Caloric Restriction	Decreased	Worse
Ramírez-López MT, Vázquez M, Bindila L, Lomazzo E, Hofmann C, Blanco RN, et al. Maternal Caloric Restriction Implemented during the Preconceptional and Pregnancy Period Alters Hypothalamic and Hippocampal Endocannabinoid Levels at Birth and Induces Overweight and Increased Adiposity at Adulthood in Male Rat Offspring. Front Behav Neurosci. 2016;10:208.	Caloric Restriction	Decreased	Worse
Rotta LN, Schmidt AP, Mello e Souza T, Nogueira CW, Souza KB, Izquierdo IA, et al. Effects of undernutrition on glutamatergic parameters in rat brain. Neurochem Res. 2003;28(8):1181-6.	Caloric Restriction	Decreased	No difference
Spencer SJ, Tilbrook A. Neonatal overfeeding alters adult anxiety and stress responsiveness. Psychoneuroendocrinology. 2009;34(8):1133-43.	Caloric Restriction	Decreased	Better
Abuaish S, Spinieli RL, McGowan PO. Perinatal high fat diet induces early activation of endocrine stress responsivity and anxiety-like behavior in neonates. Psychoneuroendocrinology. 2018;98:11-21.	Fats	Increased	Worse
Bayandor P, Farajdokht F, Mohaddes G, Diba R, Hosseindoost M, Mehri K, et al. The effect of troxerutin on anxiety- and depressive-like behaviours in the offspring of high-fat diet fed dams. Arch Physiol Biochem. 2019;125(2):156-62	Fats	Increased	Worse
Eshra MA, Rashed LA, Eltelbany RFA, Omar H, ShamsEldeen AM. Omega-3 modulates anxiety and improves autistic like features induced by high fat diet but not valproate. Neurology, Psychiatry and Brain Research. 2019;33:11-21.	Fats	Increased	Worse
Fernandes C, Grayton H, Poston L, Samuelsson AM, Taylor PD, Collier DA, et al. Prenatal exposure to maternal obesity leads to hyperactivity in offspring. Molecular Psychiatry. 2012;17(12):1159-60.	Fats	Increased	No difference
Gawlińska K, Gawliński D, Korostyński M, Borczyk M, Frankowska M, Piechota M, et al. Maternal dietary patterns are associated with susceptibility to a depressive-like phenotype in rat offspring. Dev Cogn Neurosci. 2021;47:100879.	Fats	Increased	Better
Glendining KA, Fisher LC, Jasoni CL. Maternal high fat diet alters offspring epigenetic regulators, amygdala glutamatergic profile and anxiety. Psychoneuroendocrinology. 2018;96:132-41.	Fats	Increased	Worse

(*Continued*)

(*Continued*)

Johnson SA, Javurek AB, Painter MS, Murphy CR, Conard CM, Gant KL, et al. Effects of a maternal high-fat diet on offspring behavioral and metabolic parameters in a rodent model. J Dev Orig Health Dis. 2017;8(1):75-88.	Fats	Increased	Worse
Kang SS, Kurti A, Fair DA, Fryer JD. Dietary intervention rescues maternal obesity induced behavior deficits and neuroinflammation in offspring. J Neuroinflammation. 2014;11:156.	Fats	Increased	Worse
Peleg-Raibstein D, Luca E, Wolfrum C. Maternal high-fat diet in mice programs emotional behavior in adulthood. Behav Brain Res. 2012;233(2):398-404.	Fats	Increased	Worse
Rincel M, Lépinay AL, Delage P, Fioramonti J, Théodorou VS, Layé S, et al. Maternal high-fat diet prevents developmental programming by early-life stress. Transl Psychiatry. 2016;6(11):e966.	Fats	Increased	Better
Rodriguez JS, Rodríguez-González GL, Reyes-Castro LA, Ibáñez C, Ramírez A, Chavira R, et al. Maternal obesity in the rat programs male offspring exploratory, learning and motivation behavior: prevention by dietary intervention pre-gestation or in gestation. Int J Dev Neurosci. 2012;30(2):75-81.	Fats	Increased	Worse
Sasaki A, de Vega WC, St-Cyr S, Pan P, McGowan PO. Perinatal high fat diet alters glucocorticoid signaling and anxiety behavior in adulthood. Neuroscience. 2013;240:1-12.	Fats	Increased	Worse
Souto TdS, Nakao FSN, Giriko CÁ, Dias CT, Cheberle AIdP, Lambertucci RH, et al. Lard-rich and canola oil-rich high-fat diets during pregnancy promote rats' offspring neurodevelopmental delay and behavioral disorders. Physiology & Behavior. 2020;213:112722.	Fats	Increased	Worse
Sullivan EL, Riper KM, Lockard R, Valleau JC. Maternal high-fat diet programming of the neuroendocrine system and behavior. Horm Behav. 2015;76:153-61.	Fats	Increased	worse
Sussman D, Germann J, Henkelman M. Gestational ketogenic diet programs brain structure and susceptibility to depression & anxiety in the adult mouse offspring. Brain Behav. 2015;5(2):e00300.	Fats	Increased	Better
Thompson JR, Valleau JC, Barling AN, Franco JG, DeCapo M, Bagley JL, et al. Exposure to a High-Fat Diet during Early Development Programs Behavior and Impairs the Central Serotonergic System in Juvenile Non-Human Primates. Front Endocrinol (Lausanne). 2017;8:164.	Fats	Increased	Worse
Winther G, Elfving B, Müller HK, Lund S, Wegener G. Maternal High-fat Diet Programs Offspring Emotional Behavior in Adulthood. Neuroscience. 2018;388:87-101.	Fats	Increased	Worse
Winther G, Eskelund A, Bay-Richter C, Elfving B, Müller HK, Lund S, et al. Grandmaternal high-fat diet primed anxiety-like behaviour in the second-generation female offspring. Behav Brain Res. 2019;359:47-55.	Fats	Increased	Worse
Wise LM, Hernández-Saavedra D, Boas SM, Pan YX, Juraska JM. Perinatal High-Fat Diet and Bisphenol A: Effects on	Fats	Increased	No difference

(*Continued*)

(*Continued*)

Behavior and Gene Expression in the Medial Prefrontal Cortex. Dev Neurosci. 2019;41(1-2):1-16.			
Zieba J, Uddin GM, Youngson NA, Karl T, Morris MJ. Long-term behavioural effects of maternal obesity in C57BL/6J mice. Physiol Behav. 2019;199:306-13.	Fats	Increased	No difference
Auguste S, Sharma S, Fisette A, Fernandes MF, Daneault C, Des Rosiers C, et al. Perinatal deficiency in dietary omega-3 fatty acids potentiates sucrose reward and diet-induced obesity in mice. Int J Dev Neurosci. 2018;64:8-13.	Fatty acids (omega 3)	Decreased	Worse
Bhatia HS, Agrawal R, Sharma S, Huo YX, Ying Z, Gomez-Pinilla F. Omega-3 fatty acid deficiency during brain maturation reduces neuronal and behavioral plasticity in adulthood. PLoS One. 2011;6(12):e28451.	Fatty acids (omega 3)	Decreased	Worse
Chen HF, Su HM. Exposure to a maternal n-3 fatty acid-deficient diet during brain development provokes excessive hypothalamic-pituitary-adrenal axis responses to stress and behavioral indices of depression and anxiety in male rat offspring later in life. J Nutr Biochem. 2013;24(1):70-80.	Fatty acids (omega 3)	Decreased	Worse
Ferraz AC, Kiss A, Araújo RL, Salles HM, Naliwaiko K, Pamplona J, et al. The antidepressant role of dietary long-chain polyunsaturated n-3 fatty acids in two phases in the developing brain. Prostaglandins Leukot Essent Fatty Acids. 2008;78(3):183-8.	Fatty acids (omega 3)	Increased	No difference
Cinquina V, Calvigioni D, Farlik M, Halbritter F, Fife-Gernedl V, Shirran SL, et al. Life-long epigenetic programming of cortical architecture by maternal 'Western' diet during pregnancy. Mol Psychiatry. 2020;25(1):22-36.	Fatty acids (omega 6)	Increased	Worse
Jones KL, Will MJ, Hecht PM, Parker CL, Beversdorf DQ. Maternal diet rich in omega-6 polyunsaturated fatty acids during gestation and lactation produces autistic-like sociability deficits in adult offspring. Behav Brain Res. 2013;238:193-9.	Fatty acids (omega 6)	Increased	Worse
Morgese MG, Tucci P, Mhillaj E, Bove M, Schiavone S, Trabace L, et al. Lifelong Nutritional Omega-3 Deficiency Evokes Depressive-Like State Through Soluble Beta Amyloid. Mol Neurobiol. 2017;54(3):2079-89.	Fatty acids (omega 6)	Increased	Worse
Palsdottir V, Månsson JE, Blomqvist M, Egecioglu E, Olsson B. Long-term effects of perinatal essential fatty acid deficiency on anxiety-related behavior in mice. Behav Neurosci. 2012;126(2):361-9.	Fatty acids (omega 6)	Increased	Worse
Queiroz MP, Lima MDS, Barbosa MQ, de Melo M, Bertozzo C, de Oliveira MEG, et al. Effect of Conjugated Linoleic Acid on Memory and Reflex Maturation in Rats Treated During Early Life. Front Neurosci. 2019;13:370.	Fatty acids (omega 6)	Increased	No difference
Sakayori N, Kikkawa T, Tokuda H, Kiryu E, Yoshizaki K, Kawashima H, et al. Maternal dietary imbalance between omega-6 and omega-3 polyunsaturated fatty acids impairs neocortical development via epoxy metabolites. Stem Cells. 2016;34(2):470-82.	Fatty acids (omega 6)	Increased	Worse

(*Continued*)

(*Continued*)

Soares JKB, de Melo APR, Medeiros MC, Queiroga RCRE, Bomfim MAD, Santiago ECA, et al. Anxiety behavior is reduced, and physical growth is improved in the progeny of rat dams that consumed lipids from goat milk: An elevated plus maze analysis. Neuroscience Letters. 2013;552:25-9.	Fatty acids (omega 6)	Increased	Better
Wainwright PE, Huang YS, DeMichele SJ, Xing H, Liu JW, Chuang LT, et al. Effects of high-gamma-linolenic acid canola oil compared with borage oil on reproduction, growth, and brain and behavioral development in mice. Lipids. 2003;38(2):171-8.	Fatty acids (omega 6)	Increased	No difference
Konycheva G, Dziadek MA, Ferguson LR, Krägeloh CU, Coolen MW, Davison M, et al. Dietary methyl donor deficiency during pregnancy in rats shapes learning and anxiety in offspring. Nutr Res. 2011;31(10):790-804.	Methyl donor	Decreased	Worse
McCoy CR, Jackson NL, Brewer RL, Moughnyeh MM, Smith DL, Jr., Clinton SM. A paternal methyl donor depleted diet leads to increased anxiety- and depression-like behavior in adult rat offspring. Biosci Rep. 2018;38(4).	Methyl donor	Decreased	Worse
Plyusnina IZ, Os'kina IN, Shchepina OA, Prasolova LA, Trut LN. A maternal methyl-containing diet alters learning ability in the Morris swimming test in adult rats. Neurosci Behav Physiol. 2007;37(5):425-8.	Methyl donor	Increased	No difference
Eseh R, Zimmerberg B. Age-dependent effects of gestational and lactational iron deficiency on anxiety behavior in rats. Behav Brain Res. 2005;164(2):214-21.	Mineral (iron)	Decreased	Worse
Fiset C, Rioux FM, Surette ME, Fiset S. Prenatal Iron Deficiency in Guinea Pigs Increases Locomotor Activity but Does Not Influence Learning and Memory. PLoS One. 2015;10(7):e0133168.	Mineral (iron)	Decreased	Worse
Laureano-Melo R, Império GE, da Silva-Almeida C, Kluck GE, Cruz Seara Fde, da Rocha FF, et al. Sodium selenite supplementation during pregnancy and lactation promotes anxiolysis and improves mnemonic performance in wistar rats' offspring. Pharmacology Biochemistry and Behavior. 2015;138:123–32.	Mineral (selenium)	Increased	Better
Schlegel RN, Spiers JG, Moritz KM, Cullen CL, Björkman ST, Paravicini TM. Maternal hypomagnesemia alters hippocampal NMDAR subunit expression and programs anxiety-like behaviour in adult offspring. Behavioural Brain Research. 2017;328:39-47.	Mineral (magnesium)	Decreased	Worse
Summers BL, Henry CM, Rofe AM, Coyle P. Dietary zinc supplementation during pregnancy prevents spatial and object recognition memory impairments caused by early prenatal ethanol exposure. Behav Brain Res. 2008;186(2):230-8.	Mineral (zinc)	Increased	No difference
Ardais AP, Rocha AS, Borges MF, Fioreze GT, Sallaberry C, Mioranzza S, et al. Caffeine exposure during rat brain development causes memory impairment in a sex selective	Phytochemical (caffeine)	Increased	Better

(*Continued*)

(*Continued*)

manner that is offset by caffeine consumption throughout life. Behav Brain Res. 2016;303:76-84.			
Bashkatova VG, Alekseeva EV, Bogdanova NG, Nazarova GA, Sudakov SK. Influence of Caffeine Consumption by Pregnant Rats on Behavior and Learning in Their Offspring. Bull Exp Biol Med. 2018;165(3):299-301.	Phytochemical (caffeine)	Increased	No difference
Hvolgaard Mikkelsen S, Obel C, Olsen J, Niclasen J, Bech BH. Maternal Caffeine Consumption during Pregnancy and Behavioral Disorders in 11-Year-Old Offspring: A Danish National Birth Cohort Study. J Pediatr. 2017;189:120-7.e1.	Phytochemical (caffeine)	Increased	Worse
Laureano-Melo R, da Silveira AL, de Azevedo Cruz Seara F, da Conceição RR, da Silva-Almeida C, Marinho BG, et al. Behavioral profile assessment in offspring of Swiss mice treated during pregnancy and lactation with caffeine. Metab Brain Dis. 2016;31(5):1071-80.	Phytochemical (caffeine)	Increased	Worse
Al-Basher GI, Aljabal H, Almeer RS, Allam AA, Mahmoud AM. Perinatal exposure to energy drink induces oxidative damage in the liver, kidney and brain, and behavioral alterations in mice offspring. Biomedicine & Pharmacotherapy. 2018;102:798-811.	Phytochemical (energy drinks)	Increased	Worse
Ward-Flanagan R, Scavuzzo C, Mandhane PJ, Bolduc FV, Dickson CT. Prenatal fruit juice exposure enhances memory consolidation in male post-weanling Sprague-Dawley rats. PLoS One. 2020;15(1):e0227938.	Phytochemical (fruit juice)	Increased	Better
Rodriguez-Gomez A, Filice F, Gotti S, Panzica G. Perinatal exposure to genistein affects the normal development of anxiety and aggressive behaviors and nitric oxide system in CD1 male mice. Physiol Behav. 2014;133:107-14.	Phytochemical (genistein)	Increased	Better
Ajarem J, Rashedi GA, Mohany M, Allam A. Neurobehavioral changes in mice offspring exposed to green tea during fetal and early postnatal development. Behavioral and Brain Functions. 2017;13(1):10.	Phytochemical (green tea)	Increased	Better
Binjumah M, Ajarem J, Ahmad M. Effects of the perinatal exposure of Gum Arabic on the development, behavior and biochemical parameters of mice offspring. Saudi J Biol Sci. 2018;25(7):1332-8.	Phytochemical (gum arabic)	Increased	Better
Toumi ML, Merzoug S, Baudin B, Tahraoui A. Quercetin alleviates predator stress-induced anxiety-like and brain oxidative signs in pregnant rats and immune count disturbance in their offspring. Pharmacology Biochemistry and Behavior. 2013;107:1-10.	Phytochemical (quercetin)	Increased	Better
Toumi ML, Merzoug S, Tahraoui A. Effects of quercetin on predator stress-related hematological and behavioral alterations in pregnant rats and their offspring. J Biosci. 2016;41(2):237-49.	Phytochemical (quercetin)	Increased	Better
Szklany K, Wopereis H, de Waard C, van Wageningen T, An R, van Limpt K, et al. Supplementation of dietary non-digestible oligosaccharides from birth onwards improve	Prebiotic	Increased	Better

(*Continued*)

(*Continued*)

social and reduce anxiety-like behaviour in male BALB/c mice. Nutr Neurosci. 2020;23(11):896-910.			
Laureano-Melo R, Caldeira RF, Guerra AF, Conceição RRd, Souza JSd, Giannocco G, et al. Maternal supplementation with Lactobacillus paracasei DTA 83 alters emotional behavior in Swiss mice offspring. PharmaNutrition. 2019;8:100148.	Probiotic	Increased	Better
Almeida SS, Garcia RA, de Oliveira LM. Effects of early protein malnutrition and repeated testing upon locomotor and exploratory behaviors in the elevated plus-maze. Physiol Behav. 1993;54(4):749-52.	Protein	Decreased	Worse
Almeida SS, Tonkiss J, Galler JR. Prenatal protein malnutrition affects exploratory behavior of female rats in the elevated plus-maze test. Physiol Behav. 1996;60(2):675-80.	Protein	Decreased	Better
Batista TH, Veronesi VB, Ribeiro ACAF, Giusti-Paiva A, Vilela FC. Protein malnutrition during pregnancy alters maternal behavior and anxiety-like behavior in offspring. Nutritional Neuroscience. 2017;20(8):437-42.	Protein	Decreased	Worse
Belluscio LM, Berardino BG, Ferroni NM, Ceruti JM, Cánepa ET. Early protein malnutrition negatively impacts physical growth and neurological reflexes and evokes anxiety and depressive-like behaviors. Physiol Behav. 2014;129:237-54.	Protein	Decreased	Worse
Crossland RF, Balasa A, Ramakrishnan R, Mahadevan SK, Fiorotto ML, Van den Veyver IB. Chronic Maternal Low-Protein Diet in Mice Affects Anxiety, Night-Time Energy Expenditure and Sleep Patterns, but Not Circadian Rhythm in Male Offspring. PLoS One. 2017;12(1):e0170127.	Protein	Decreased	Worse
da Silva Hernandes A, Françolin-Silva AL, Valadares CT, Fukuda MT, Almeida SS. Effects of different malnutrition techniques on the behavior of rats tested in the elevated T-maze. Behav Brain Res. 2005;162(2):240-5.	Protein	Decreased	Better
Françolin-Silva AL, da Silva Hernandes A, Fukuda MT, Valadares CT, Almeida SS. Anxiolytic-like effects of short-term postnatal protein malnutrition in the elevated plus-maze test. Behav Brain Res. 2006;173(2):310-4.	Protein	Decreased	Better
Furuse T, Miyake K, Kohda T, Kaneda H, Hirasawa T, Yamada I, et al. Protein-restricted diet during pregnancy after insemination alters behavioral phenotypes of the progeny. Genes Nutr. 2017;12:1.	Protein	Decreased	Worse
	Vitamin (folic acid)	Increased	
Galler JR, Bryce CP, Zichlin ML, Waber DP, Exner N, Fitzmaurice GM, et al. Malnutrition in the first year of life and personality at age 40. J Child Psychol Psychiatry. 2013;54(8):911-9.	Protein	Decreased	Worse
Hernandes AS, Almeida SS. Postnatal protein malnutrition affects inhibitory avoidance and risk assessment behaviors in two models of anxiety in rats. Nutr Neurosci. 2003;6(4):213-9.	Protein	Decreased	Better

(*Continued*)

(*Continued*)

Lotufo BM, Tenório F, Barradas PC, Guedes PL, Lima SS, Rocha MLM, et al. Maternal protein-free diet during lactation programs male Wistar rat offspring for increased novelty-seeking, locomotor activity, and visuospatial performance. Behav Neurosci. 2018;132(2):114-27.	Protein	Decreased	No difference
Nagamachi S, Nishigawa T, Takakura M, Ikeda H, Kodaira M, Yamaguchi T, et al. Dietary L-serine modifies free amino acid composition of maternal milk and lowers the body weight of the offspring in mice. J Vet Med Sci. 2018;80(2):235-41.	Protein	Increased	No difference
Naik AA, Patro IK, Patro N. Slow Physical Growth, Delayed Reflex Ontogeny, and Permanent Behavioral as Well as Cognitive Impairments in Rats Following Intra-generational Protein Malnutrition. Front Neurosci. 2015;9:446.	Protein	Decreased	Worse
Nätt D, Barchiesi R, Murad J, Feng J, Nestler EJ, Champagne FA, et al. Perinatal Malnutrition Leads to Sexually Dimorphic Behavioral Responses with Associated Epigenetic Changes in the Mouse Brain. Sci Rep. 2017;7(1):11082.	Protein	Decreased	Worse
Pereira-da-Silva MS, Cabral-Filho JE, de-Oliveira LM. Effect of early malnutrition and environmental stimulation in the performance of rats in the elevated plus maze. Behav Brain Res. 2009;205(1):286-9.	Protein	Decreased	Better
Pillay N, Rimbach R, Rymer T. Pre- and postnatal dietary protein deficiency influences anxiety, memory and social behaviour in the African striped mouse Rhabdomys dilectus chakae. Physiol Behav. 2016;161:38-46.	Protein	Decreased	Worse
Reyes-Castro LA, Rodriguez JS, Charco R, Bautista CJ, Larrea F, Nathanielsz PW, et al. Maternal protein restriction in the rat during pregnancy and/or lactation alters cognitive and anxiety behaviors of female offspring. Int J Dev Neurosci. 2012;30(1):39-45.	Protein	Decreased	Worse
Strata F, Giritharan G, Sebastiano FD, Piane LD, Kao C-N, Donjacour A, et al. Behavior and Brain Gene Expression Changes in Mice Exposed to Preimplantation and Prenatal Stress. Reproductive Sciences. 2015;22(1):23-30.	Protein	Increased	Mixed
	Fats	Decreased	
Torres DB, Lopes A, Rodrigues AJ, Cerqueira JJ, Sousa N, Gontijo JAR, et al. Anxiety-like behavior and structural changes of the bed nucleus of the stria terminalis (BNST) in gestational protein-restricted male offspring. J Dev Orig Health Dis. 2018;9(5):536-43.	Protein	Decreased	Worse
Ware S, Voigt JP, Langley-Evans SC. Body composition and behaviour in adult rats are influenced by maternal diet, maternal age and high-fat feeding. J Nutr Sci. 2015;4:e3.	Protein	Decreased	Worse
Watkins AJ, Ursell E, Panton R, Papenbrock T, Hollis L, Cunningham C, et al. Adaptive responses by mouse early embryos to maternal diet protect fetal growth but predispose to adult onset disease. Biol Reprod. 2008;78(2):299-306.	Protein	Decreased	Worse
Watkins AJ, Wilkins A, Cunningham C, Perry VH, Seet MJ, Osmond C, et al. Low protein diet fed exclusively during	Protein	Decreased	Worse

(*Continued*)

(*Continued*)

mouse oocyte maturation leads to behavioural and cardiovascular abnormalities in offspring. J Physiol. 2008;586(8):2231-44.			
Zhang L, Guadarrama L, Corona-Morales AA, Vega-Gonzalez A, Rocha L, Escobar A. Rats Subjected to Extended L-Tryptophan Restriction During Early Postnatal Stage Exhibit Anxious-Depressive Features and Structural Changes. Journal of Neuropathology & Experimental Neurology. 2006;65(6):562-70.	Protein	Decreased	Worse
Bukhari SHF, Clark OE, Williamson LL. Maternal high fructose diet and neonatal immune challenge alter offspring anxiety-like behavior and inflammation across the lifespan. Life Sci. 2018;197:114-21.	Sugar	Increased	Mixed
Collison KS, Inglis A, Shibin S, Andres B, Ubungen R, Thiam J, et al. Differential effects of early-life NMDA receptor antagonism on aspartame-impaired insulin tolerance and behavior. Physiol Behav. 2016;167:209-21.	Sugar	Increased	Worse
Le Q, Li Y, Hou W, Yan B, Yu X, Song H, et al. Binge-Like Sucrose Self-Administration Experience Inhibits Cocaine and Sucrose Seeking Behavior in Offspring. Front Behav Neurosci. 2017;11:184.	Sugar	Increased	Worse
Bilbo SD, Tsang V. Enduring consequences of maternal obesity for brain inflammation and behavior of offspring. Faseb j. 2010;24(6):2104-15.	Trans Fat	Increased	Worse
	Fats		
Pase CS, Roversi K, Trevizol F, Roversi K, Kuhn FT, Schuster AJ, et al. Influence of perinatal trans fat on behavioral responses and brain oxidative status of adolescent rats acutely exposed to stress. Neuroscience. 2013;247:242-52.	Trans Fat	Increased	Worse
Roversi K, Pase CS, Roversi K, Vey LT, Dias VT, Metz VG, et al. Trans fat intake across gestation and lactation increases morphine preference in females but not in male rats: Behavioral and biochemical parameters. Eur J Pharmacol. 2016;788:210-7.	Trans Fat	Increased	Better
Ear PH, Chadda A, Gumusoglu SB, Schmidt MS, Vogeler S, Malicoat J, et al. Maternal Nicotinamide Riboside Enhances Postpartum Weight Loss, Juvenile Offspring Development, and Neurogenesis of Adult Offspring. Cell Rep. 2019;26(4):969-83.e4.	Vitamin (B3)	Increased	Better
Wu YC, Wang YJ, Tseng GF. Ascorbic acid and α-tocopherol supplement starting prenatally enhances the resistance of nucleus tractus solitarius neurons to hypobaric hypoxic challenge. Brain Struct Funct. 2011;216(2):105-22.	Vitamin (C)	Increased	Better
Glenn MJ, Adams RS, McClurg L. Supplemental dietary choline during development exerts antidepressant-like effects in adult female rats. Brain Res. 2012;1443:52-63.	Vitamin (choline)	Increased	Better
Langley EA, Krykbaeva M, Blusztajn JK, Mellott TJ. High maternal choline consumption during pregnancy and nursing alleviates deficits in social interaction and improves	Vitamin (choline)	Increased	Better

(*Continued*)

(*Continued*)

anxiety-like behaviors in the BTBR T+Itpr3tf/J mouse model of autism. Behav Brain Res. 2015;278:210-20.			
Sahara Y, Matsuzawa D, Ishii D, Fuchida T, Goto T, Sutoh C, et al. Paternal methyl donor deficient diets during development affect male offspring behavior and memory-related gene expression in mice. Dev Psychobiol. 2019;61(1):17-28.	Vitamin (choline and folic acid)	Decreased	Worse
Schulz KM, Pearson JN, Gasparrini ME, Brooks KF, Drake-Frazier C, Zajkowski ME, et al. Dietary choline supplementation to dams during pregnancy and lactation mitigates the effects of in utero stress exposure on adult anxiety-related behaviors. Behav Brain Res. 2014;268:104-10.	Vitamin (choline)	Increased	Better
Harms LR, Eyles DW, McGrath JJ, Mackay-Sim A, Burne TH. Developmental vitamin D deficiency alters adult behaviour in 129/SvJ and C57BL/6J mice. Behav Brain Res. 2008;187(2):343-50.	Vitamin (D)	Decreased	No difference
Pan P, Jin DH, Chatterjee-Chakraborty M, Halievski K, Lawson D, Remedios D, et al. The effects of vitamin D_3 during pregnancy and lactation on offspring physiology and behavior in sprague-dawley rats. Dev Psychobiol. 2014;56(1):12-22.	Vitamin (D)	Increased	Better
Vuillermot S, Luan W, Meyer U, Eyles D. Vitamin D treatment during pregnancy prevents autism-related phenotypes in a mouse model of maternal immune activation. Molecular Autism. 2017;8.	Vitamin (D)	Increased	Better
Ambrogini P, Ciuffoli S, Lattanzi D, Minelli A, Bucherelli C, Baldi E, et al. Maternal dietary loads of α-tocopherol differentially influence fear conditioning and spatial learning in adult offspring. Physiol Behav. 2011;104(5):809-15.	Vitamin (E)	Increased	No difference
Desrumaux CM, Mansuy M, Lemaire S, Przybilski J, Le Guern N, Givalois L, et al. Brain Vitamin E Deficiency During Development Is Associated With Increased Glutamate Levels and Anxiety in Adult Mice. Front Behav Neurosci. 2018;12:310.	Vitamin (E)	Increased	Better
Barua S, Chadman KK, Kuizon S, Buenaventura D, Stapley NW, Ruocco F, et al. Increasing maternal or post-weaning folic acid alters gene expression and moderately changes behavior in the offspring. PLoS One. 2014;9(7):e101674.	Vitamin (folic acid)	Increased	Worse
Ferguson SA, Berry KJ, Hansen DK, Wall KS, White G, Antony AC. Behavioral effects of prenatal folate deficiency in mice. Birth Defects Res A Clin Mol Teratol. 2005;73(4):249-52.	Vitamin (folic acid)	Decreased	Worse
Yan Z, Jiao F, Yan X, Ou H. Maternal Chronic Folate Supplementation Ameliorates Behavior Disorders Induced by Prenatal High-Fat Diet Through Methylation Alteration of BDNF and Grin2b in Offspring Hippocampus. Molecular Nutrition & Food Research. 2017;61(12):1700461.	Vitamin (folic acid)	Increased	Better
Yang X, Sun W, Wu Q, Lin H, Lu Z, Shen X, et al. Excess Folic Acid Supplementation before and during Pregnancy and Lactation Alters Behaviors and Brain Gene Expression in Female Mouse Offspring. Nutrients. 2021;14(1).	Vitamin (folic acid)	Increased	Better

(*Continued*)

(*Continued*)

Guedine CRC, Pordeus LCM, Riul TR, Jordão AAJ, Almeida SS. Cafeteria diet during lactation and/or post-lactation altered lipid profile/lipid peroxidation and increased anxiety-like behavior in male rat offspring. Nutr Neurosci. 2020;23(7):526-36.	Western Diet	Increased	Worse
Hiramatsu L, Kay JC, Thompson Z, Singleton JM, Claghorn GC, Albuquerque RL, et al. Maternal exposure to Western diet affects adult body composition and voluntary wheel running in a genotype-specific manner in mice. Physiol Behav. 2017;179:235-45.	Western Diet	Increased	Worse
Marcolin Mde L, Benitz Ade N, Arcego DM, Noschang C, Krolow R, Dalmaz C. Effects of early life interventions and palatable diet on anxiety and on oxidative stress in young rats. Physiol Behav. 2012;106(4):491-8.	Western Diet	Increased	Better
Shalev U, Tylor A, Schuster K, Frate C, Tobin S, Woodside B. Long-term physiological and behavioral effects of exposure to a highly palatable diet during the perinatal and post-weaning periods. Physiol Behav. 2010;101(4):494-502.	Western Diet	Increased	No difference
Speight A, Davey WG, McKenna E, Voigt JW. Exposure to a maternal cafeteria diet changes open-field behaviour in the developing offspring. Int J Dev Neurosci. 2017;57:34-40.	Western Diet	Increased	Better
Thompson JR, Gustafsson HC, DeCapo M, Takahashi DL, Bagley JL, Dean TA, et al. Maternal Diet, Metabolic State, and Inflammatory Response Exert Unique and Long-Lasting Influences on Offspring Behavior in Non-Human Primates. Front Endocrinol (Lausanne). 2018;9:161.	Western Diet	Increased	Worse
Wright T, Langley-Evans SC, Voigt JP. The impact of maternal cafeteria diet on anxiety-related behaviour and exploration in the offspring. Physiol Behav. 2011;103(2):164-72.	Western Diet	Increased	Better
